# scKWARN: Kernel-weighted-average robust normalization for single-cell RNA-seq data

**DOI:** 10.1093/bioinformatics/btae008

**Published:** 2024-01-17

**Authors:** Chih-Yuan Hsu, Chia-Jung Chang, Qi Liu, Yu Shyr

**Affiliations:** Department of Biostatistics, Vanderbilt University Medical Center, Nashville, TN 37203, United States; Center for Quantitative Sciences, Vanderbilt University Medical Center, Nashville, TN 37203, United States; Department of Biostatistics, Vanderbilt University Medical Center, Nashville, TN 37203, United States; Center for Quantitative Sciences, Vanderbilt University Medical Center, Nashville, TN 37203, United States; Department of Biomedical Engineering, National Cheng Kung University, Tainan 701, Taiwan; Department of Biostatistics, Vanderbilt University Medical Center, Nashville, TN 37203, United States; Center for Quantitative Sciences, Vanderbilt University Medical Center, Nashville, TN 37203, United States; Department of Biostatistics, Vanderbilt University Medical Center, Nashville, TN 37203, United States; Center for Quantitative Sciences, Vanderbilt University Medical Center, Nashville, TN 37203, United States

## Abstract

**Motivation:**

Single-cell RNA-seq normalization is an essential step to correct unwanted biases caused by sequencing depth, capture efficiency, dropout, and other technical factors. Existing normalization methods primarily reduce biases arising from sequencing depth by modeling count-depth relationship and/or assuming a specific distribution for read counts. However, these methods may lead to over or under-correction due to presence of technical biases beyond sequencing depth and the restrictive assumption on models and distributions.

**Results:**

We present scKWARN, a Kernel Weighted Average Robust Normalization designed to correct known or hidden technical confounders without assuming specific data distributions or count-depth relationships. scKWARN generates a pseudo expression profile for each cell by borrowing information from its fuzzy technical neighbors through a kernel smoother. It then compares this profile against the reference derived from cells with the same bimodality patterns to determine the normalization factor. As demonstrated in both simulated and real datasets, scKWARN outperforms existing methods in removing a variety of technical biases while preserving true biological heterogeneity.

**Availability and Implementation:**

scKWARN is freely available at https://github.com/cyhsuTN/scKWARN.

## 1 Introduction

Providing transcriptomes at the resolution of individual cells, single-cell RNA sequencing (scRNAseq) has become a powerful tool for identifying and characterizing cellular heterogeneity and transition states (Han *et al.* 2018, Lee *et al.* 2020, Shami *et al.* 2020). However, scRNAseq data have high level of technical noise and sparsity due to complicated and multi-step experimental protocols and tiny amount of starting material per cell, which make the analysis very challenging. Normalization is the first essential step that adjusts the effect of technical biases to make expression comparable across genes and/or cells. Accurate normalization is critical for valid and biologically meaningful downstream analysis.

There are a number of normalization methods available for bulk RNA-seq data. They generally calculate global scaling factors based on the non-DE assumption, that is, most of genes are not differentially expressed (DE) across experiments. The most commonly used approaches, such as Trimmed Mean of M-values (TMM) in edgeR (Robinson and Oshlack 2010), Relative Log Expression (RLE) in DESeq2 (Love *et al.* 2014), and Median Ratio Normalization (MRN) (Maza *et al.* 2013), achieve great performance. However, they are not applicable in the scRNAseq setting due to its characteristics of excessive number of zeros. The extreme sparsity would result in a geometric mean of zero for RLE and undefined M values for TMM. The unbalanced sizes between rare and abundant cell populations in scRNAseq would partially violate the non-DE assumption and introduce spurious biases.

Recently, several methods tailored for scRNAseq normalization have emerged. They generally fall into two categories, global and gene-specific scaling factors. Global scaling factors correct cell-specific systematic biases, meaning that genes are normalized by the same constant in each cell. The most straightforward and widely-used approach is RC (relative counts), which divides raw counts by the total number of counts in each cell, then multiplies a constant factor (Hafemeister and Satija 2019). If RNA composition is different across cells, the usage of spike in RNAs instead of library sizes might be better to estimate scale factors, such as BASiCS (Vallejos *et al.* 2015), SAMstrt (Katayama *et al.* 2013) and Gamma Regression Model (GRM) (Ding *et al.* 2015). To reduce the effect of problematic zeros, scran (Lun *et al.* 2016) sums expression across pools of cells by library sizes, normalizes the cell pool against an average reference, and deconvolves the pool-based size factors to yield cell-based factors by a linear system. Assuming single-cell RNAseq data follow the Pareto distribution, PsiNorm (Borella *et al.* 2021) derives a global normalization factor from the Pareto distribution shape parameter that is inversely proportion to the sequencing depth. Unlike global scaling factors, which assume uniform technical biases across all genes in each cell, gene-specific scaling methods acknowledge diverse relationships between expression and technical factors across genes (Bacher *et al.* 2017, Hafemeister and Satija 2019). For example, SCnorm (Bacher *et al.* 2017) groups genes by their similarity on count-depth relationships and applies linear quantile regression to determine scale factors within each group. sctransform (Hafemeister and Satija 2019) utilizes a linear negative binomial regression model to estimate the count-depth relationship for each gene. It predicts expected counts using stable coefficient estimates from a kernel smoother and considers the residuals from the model as normalized expression levels. While these pioneering approaches successfully address noise arising from sequencing depth, their performance relies on the assumption of a faithful linear count-depth relationship and a specific distribution of read counts. The count-depth relationships for specific marker genes, which are highly expressed in one cell type and not expressed in others, can vary significantly across cell populations. Assuming uniform count-depth relationships across cells may introduce artifacts during normalization. Moreover, while sequencing depth is a major source of technical noise, other factors such as amplification bias, capture efficiency, and dropout rates, all contribute to unwanted expression variations, which need to be adjusted as well.

In this article, we introduce scKWARN, a novel scRNAseq normalization approach without assumption on data distributions or count-depth relationships. scKWARN first generates a pseudo-expression profile for each cell by leveraging information from its fuzzy technical neighbors. It then compares the pseudo profile against a reference derived from cells with the similar bimodality patterns to estimate the normalization factor based on the non-DE assumption. By defining technical neighbors through the similarity of non-zero counts distribution, scKWARN inherently consider any technical factors contributing to unwanted expression variation. Cells with similar bimodality patterns are highly likely to be from the same cell population, ensuring the non-DE assumption remains valid even in the presence of extremely unbalanced cell populations. Simulated and real case studies demonstrated that scKWARN is robust against a variety of technical and biological factors, with its performance independent of data distributions and count-depth relationships.

## 2 Methods

**Figure 1.**
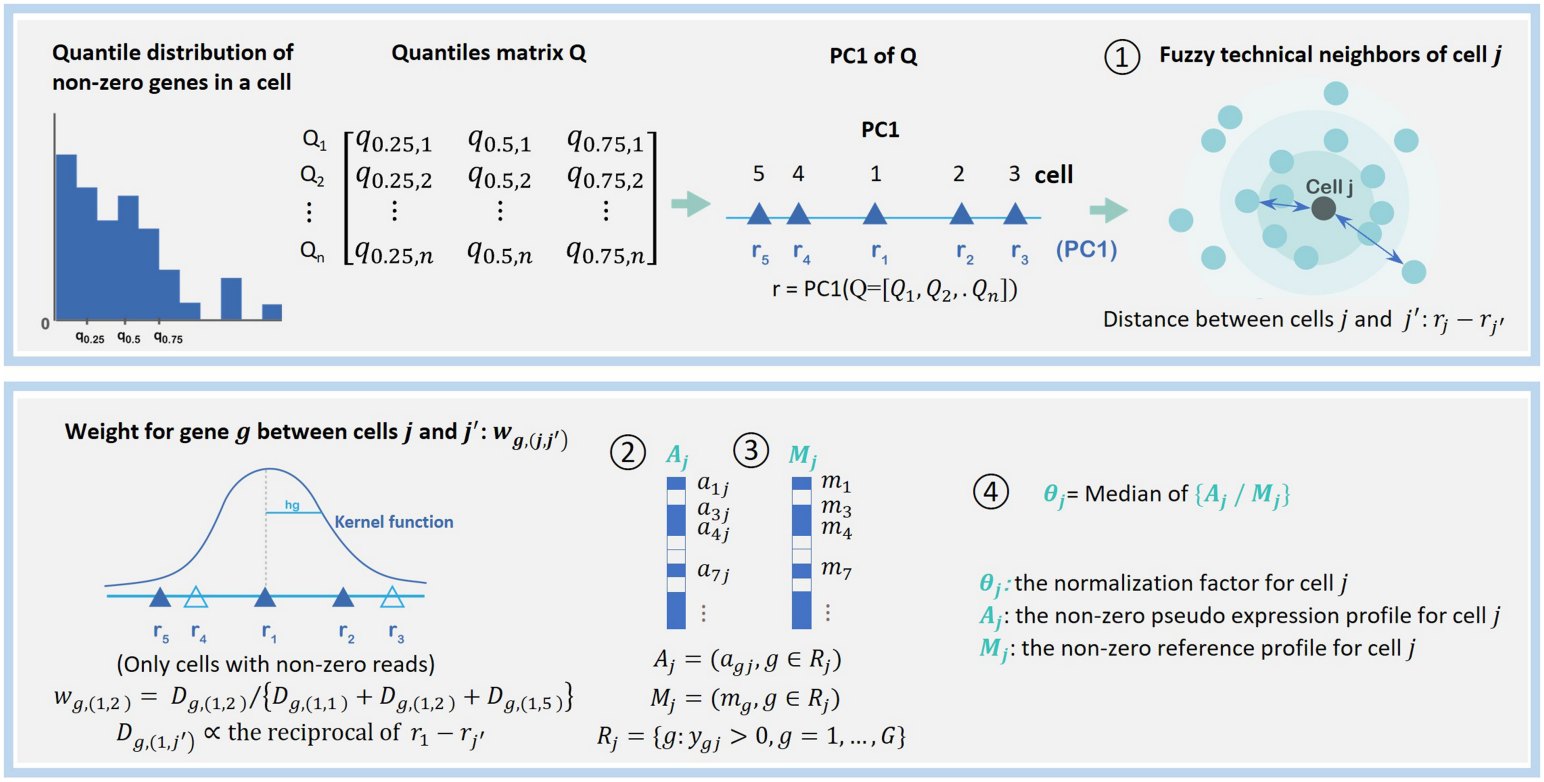
Overview of scKWARN. Cell distances are calculated based on the first principal component of the 25th, 50th, and 75th percentiles of non-zero profiles. The non-zero pseudo profile of each cell is generated by summing weighted expression of its neighbors via a kernel smoother. The normalization factor of each cell is determined by the median ratio of its pseudo profile against its non-zero reference profile.

scKWARN, single-cell Kernel-Weighted-Average Robust Normalization, consists of four steps (Fig. 1 & Pseudocode in Supplementary Materials).

The first step is to define technical neighbors, ie, cells that share similar technical factors such as library sizes or dropout rates. This concept is motivated by scran (Lun *et al.* 2016), which pools cells with similar library sizes. Technical similarity is roughly calculated based on the first principal component of the 25th, 50th, and 75th percentiles of non-zero expression in cells. Suppose that scRNAseq data involve G genes and n cells. $y_{gj}$ denotes the read count of gene g in the cell j, g=1,…,G and j=1,…,n. $R_{j}=\{g:\;y_{gj}\;>\;0,\;g=\;1,\ldots ,\;G\}$ is the subset of genes with non-zero counts in cell j. $Q_{j}=[q_{0.25,j},q_{0.50,j},q_{0.75,j}]$ of $\left\{\log(y_{gj}),\;g\in R_{j}\right\}$ is the three-dimensional vector comprising 25th, 50th, and 75th percentiles of log-transformed non-zero counts in cell j. r $=\left[r_{1},\;r_{2},\ldots r_{n}\right]=$ PC1($Q=[Q_{1},\;Q_{2},\ldots Q_{n}]$), is the first principal component of Q. The technical similarity between cells j and j′ is estimated by the distance $r_{j}-r_{j^{\prime }}$.

The second step is to generate a pseudo gene expression profile for each cell by leveraging information from its technical neighbors using a kernel smoother. In scKWARN, “fuzzy” technical neighbors are defined, indicating no hard boundary to group cells. Instead, scKWARN generates the pseudo profile for non-zero genes in each cell by summing weighted expression of its technical neighbors via a kernel smoother. This approach reduces technical variability among cells, providing a robust estimation. The weight $w_{g,(j,j^{\prime })}$ used to calculate the pseudo-profile is both cell- and gene-specific, where $w_{g,(j,j^{\prime })}=D_{g,(j,j^{\prime })}/\sum_{j^{\prime }\in C_{g}}D_{g,(j,j^{\prime })}$, $D_{g,(j,j^{\prime })}=\;K\{(r_{j}-r_{j^{\prime }})/h_{g}\}$, K(·) is the Gaussian kernel, and $h_{g}$ is the SJ bandwidth (Sheather and Jones 1991) data-driven by $\left\{r_{j},\;j\in C_{g}\right\}$. $C_{g}=\left\{j:\;y_{gj}>0,\;j=1,\ldots ,\;n\right\}$ is the subset of cells with non-zero expression of gene g. The pseudo expression profile for cell j, $A_{j}=(a_{gj},\;g\in R_{j})$, is generated from the kernel-weighted geometric means of non-zero genes of cell j, where $a_{gj}=\exp\left\{\sum_{j^{\prime }\in C_{g}}w_{g,\left(j,j^{\prime }\right)}\log\left(y_{gj^{\prime }}\right)\right\}$.

The third step is to build a reference profile for each cell. The reference profile for cell j, denoted as $M_{j}=\left(m_{g},\;g\in R_{j}\right)$, where $m_{g}=\exp\left\{\sum_{j\in C_{g}}\log\left(y_{gj}\right)/|C_{g}|\right\}$, is defined as the geometric mean of $R_{j}$ across $C_{g}$. In other words, for each non-zero gene g in cell j, the process entails finding the subset of cells expression this gene ($C_{g}$) and calculating the geometric mean of the gene expression across $C_{g}$. To be noted, $M_{j}$ is built based on non-zero genes in the cell j, therefore $M_{j}$ is specific for cell j.

The fourth step involves comparing the pseudo profile of each cell to its specific reference to determine the size factor based on the non-DE assumption. scKWARN estimates the normalization factor using a median-ratio strategy, where $\theta_{j}=\mathrm{median}\{A_{j}/M_{j}\}$. Rather than all cells sharing the same reference, each cell in scKWARN has its own reference for comparison. These references are calculated from cells with similar cell types. The reference profile for a given cell is calculated from all cells with non-zero genes detected in that cell, meaning that the reference abundances of cell-specific genes are most likely to be derived from cells belonging to the same cell type. Using scRNAseq data generated from five cell lines as an example, reference profiles of cells belonging to the same cell type are grouped together. In other words, cells from the same cell type tend to have similar reference abundances (Supplementary Fig. S1). This approach ensures that the non-DE assumption is not violated, even in cases where cell populations are extremely unbalanced.

## 3 Results

### 3.1 Performance of scKWARN on simulated data

The performance of scKWARN was evaluated and compared against five normalization methods: RC, scran, SCnorm, sctransform, and PsiNorm, using three simulated settings. The data were generated following the simulation studies described in scran (Lun *et al.* 2016), SCnorm (Bacher *et al.* 2017), and sctransform (Hafemeister and Satija 2019). In the first two simulation settings, the gene count and sequencing depth followed a linear relationship on the log-scale but with different error distributions: normal (Bacher *et al.* 2017) (Simulation I) and negative binomial distributions (Hafemeister and Satija 2019) (Simulation II), respectively. In the third simulation setting, counts were sampled from a negative binomial distribution without the assumption of count-depth relationship (Lun *et al.* 2016) (Simulation III). All three simulation studies involved 3000 genes for three subpopulations. For each simulation setting, five scenarios were designed by altering library sizes, RNA compositions (the percentage of counts from the most highly expressed genes), capture efficiency (dropout rates, the proportion of zero counts), cell population compositions, and the DE level (Details in Supplementary Materials). 100 simulation datasets were generated for each scenario.

We utilized five metrics to evaluate performance: Bias, Root Mean Square Error (RMSE), Sensitivity, Specificity, and F1 score. These measures assess the preservation of biological variability while mitigating technical noise. MAST was used to identify DE genes (Finak *et al.* 2015). In addition to measuring the difference between the true and the estimated log2-fold-change, as Bias does, RMSE also takes into account the variance of this difference. The F1 score provides a balance between precision and recall. As such, our primary focus was on RMSE and F1 score (Details in Supplementary Materials). The performance, as measured by Bias, Sensitivity, and Specificity, were illustrated in Supplementary Figs S2–S4.

**Figure 2.**
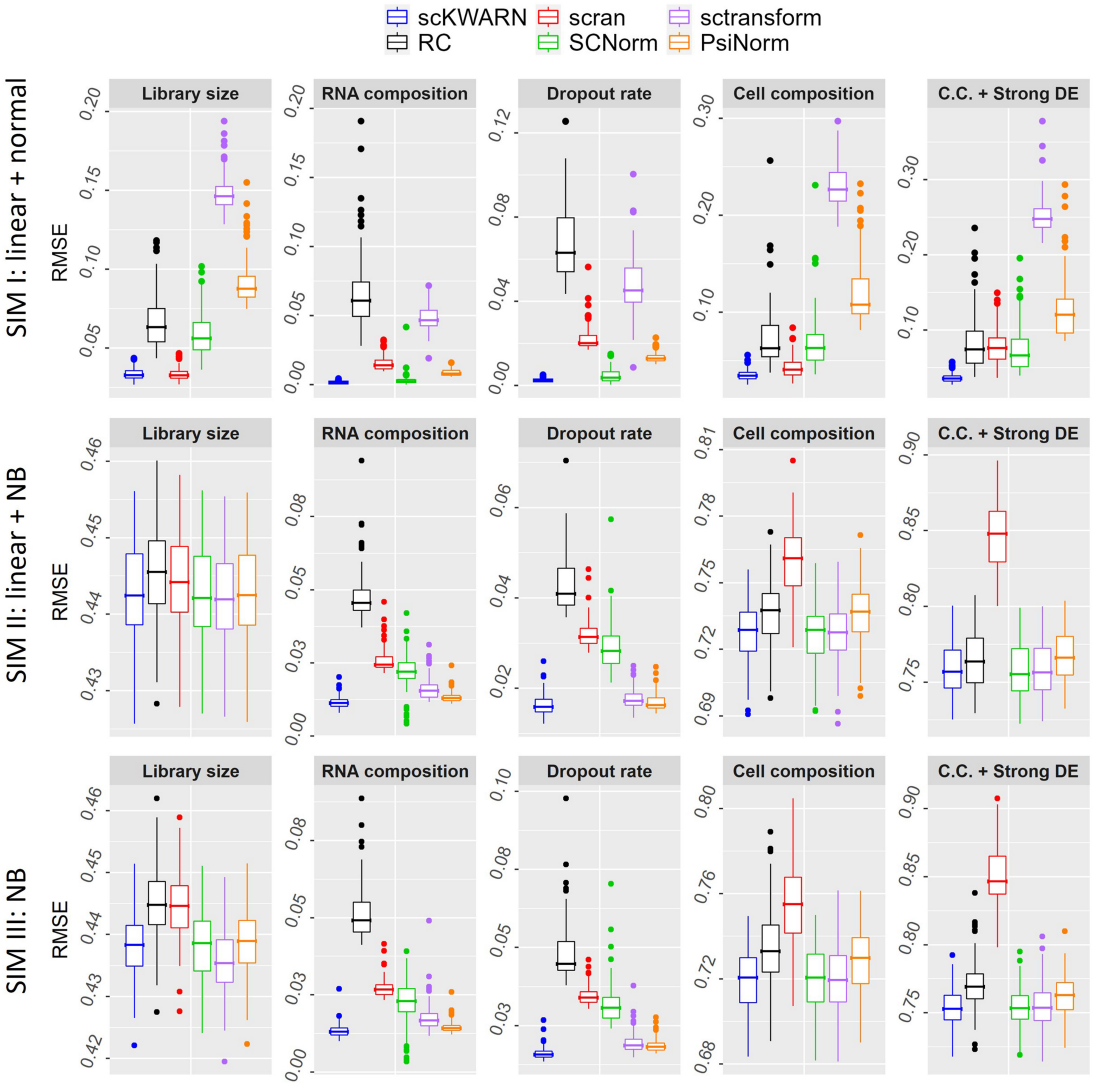
Performance comparison in terms of RMSE in three simulation settings with five scenarios. In simulation I and II (the top and middle panels), the gene count and sequencing depth both followed a linear relationship on the log-scale but with different error distributions, normal and negative binomial distributions, respectively. In simulation III (the bottom panel), counts were sampled from a negative binomial distribution without the assumption of count-depth relationship. Five scenarios were designed by altering library sizes, RNA compositions, dropout rates, cell population compositions and the DE level.

**Figure 3.**
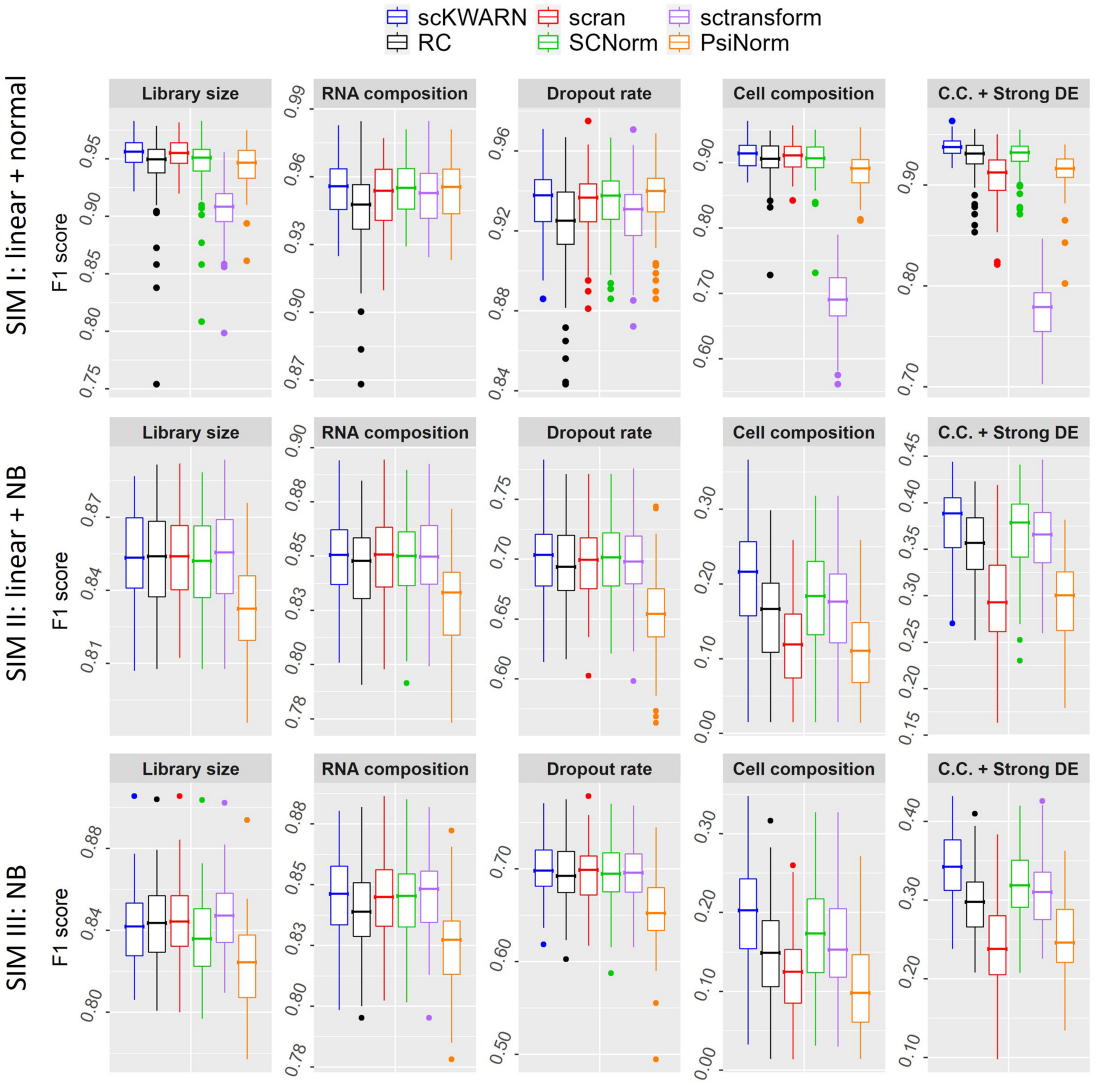
Performance comparison on F1 score in three simulation settings with five scenarios. In simulation I and II (the top and middle panels), the gene count and sequencing depth both followed a linear relationship on the log-scale but with different error distributions, normal and negative binomial distributions, respectively. In simulation III (the bottom panel), counts were sampled from a negative binomial distribution without the assumption of count-depth relationship. Five scenarios were designed by altering library sizes, RNA compositions, dropout rates, cell population compositions and the DE level.

scKWARN demonstrated robustness against technical noises introduced by library sizes, RNA composition, capture efficiency, and biological effects of unbalanced cell populations and strong DE level, which obtained the lowest RMSE (Fig. 2) and the highest F1 scores (Fig. 3) across all five scenarios in each setting. Furthermore, the performance of scKWARN is independent of model and distribution assumptions, consistently achieving the best results in terms of RMSE and F1 scores across all three settings. In comparison, RC, scran, SCnorm, sctransform, and PsiNorm worked well in specific scenarios and/or settings, but their performances compromised in others (Figs 2 and 3). RC, as anticipated, was intolerant to RNA composition and dropout biases, resulting in the highest RMSE and the lowest F1 score in these two specific scenarios across all three settings. Scran successfully corrected the effect of sequencing depth but showed limitations when addressing the effect of RNA composition and dropout rates (Figs 2 and 3). In the simulations II and III, scran exhibited high RMSE values when cell populations were unbalanced and even the highest when strong DE level were added. PsiNorm consistently achieved low RMSE values across all three settings when biases were introduced by RNA composition and dropout rates. However, it showed high RMSE values in the simulation I with the other three scenarios (library size, cell composition, and cell composition+strong DE) and low F1 scores in the simulations II and III. Both SCnorm and sctransform estimate gene-specific scaling factors, and their performances were highly dependent on whether the simulation settings fit their underlying models. SCnorm worked effectively under the linear model with a normal distribution (Simulation I), while sctransform showed good performance under the negative binomial distribution (Simulation II and III). Despite aiming to adjust only the technical bias from sequencing depth by modeling count-depth relationships, they exhibited tolerance against RNA composition and capture efficiency biases. In their appropriate settings, SCnorm and sctransform achieved lower RMSE and higher F1 values than RC and scran. Their success in removing these noises can be attributed to the usage of gene-specific scale factors, where highly and lowly expressed genes are modeled separately. It is noteworthy that scKWARN, a method estimating global scaling factors, outperformed SCnorm and sctransform even in these two scenarios (Figs 2 and 3). In summary, scKWARN demonstrated robustness against a variety of known technical factors without imposing restrictive assumptions on models or distributions.

### 3.2 Performance of scKWARN on real data

**Figure 4.**
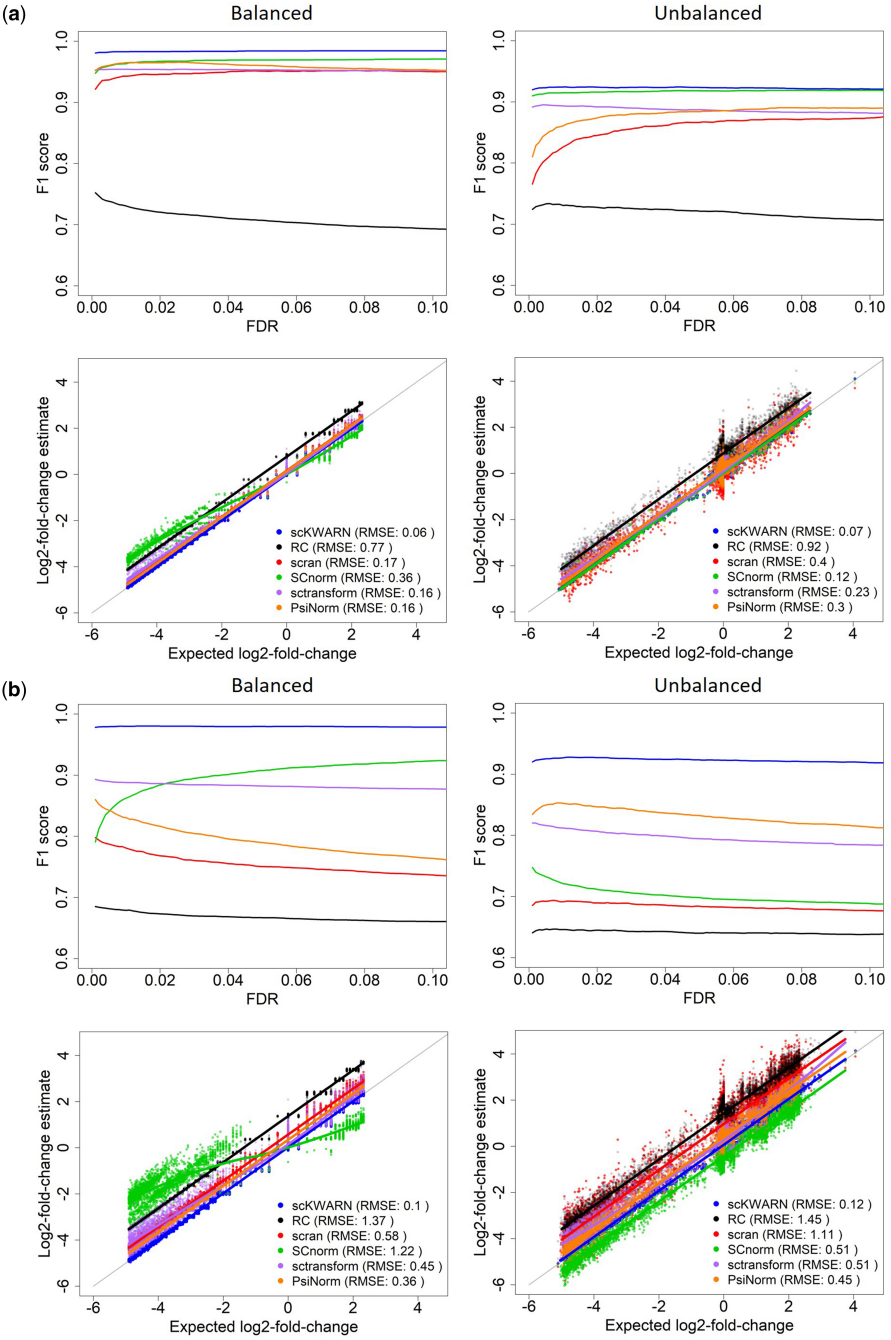
Performance comparison on the PBMC33K dataset with Moderate DE (a) and Strong DE (b) settings. The balanced setting has each cell population of 1000 cells (left panel), while the unbalanced includes one of 1000 and the other of only 100 cells (right panel).

We assessed the performance of scKWARN on two real scRNAseq datasets in terms of preserving biological variability and marker detection (Details in Supplementary Materials). The first dataset, PBMC33K, generated by 10x Genomics, comprises 33 148 human peripheral blood mononuclear cells and 32 738 genes. Similar to the simulation, we randomly extracted 1000 B cells and generated two cell populations. For each cell population, a certain percentage of genes were randomly chosen, and each gene was upsized by different factors to introduce varying magnitude of DE. We simulated four scenarios involving two factors: moderate/strong DE and balanced/unbalanced cell populations. In the moderate setting, 10% of genes were chosen randomly to be DE, while in the strong DE scenario, this percentage increased to 25%. The balanced setting involved each cell population having 1000 cells, while the unbalanced setting included one population with 1000 cells and the other with only 100 cells. We compared different normalization strategies based on the F1 score and their ability to recover the true fold change (Fig. 4). scKWARN achieved the best performance with the highest F1 scores and the lowest RMSE values in all four scenarios, accurately estimating fold changes that closely matched the true values (Fig. 4). sctransform also exhibited good performance across all settings. Conversely, RC performed poorly, displaying the lowest F1 scores and highest RMSE values, along with a shift between the true and the estimated fold changes. SCnorm, scran, and PsiNorm functioned properly when only moderate DE was present; however, their performance compromised in the presence of strong DE. SCnorm and scran’s performance deteriorated further in scenarios where strong DE and unbalanced cell population coexisted (Fig. 4b). In addition, SCnorm appeared to over-adjust genes with large magnitude of DE, resulting in an underestimation of fold changes in the balanced setting. We also introduced four scenarios where each gene was downsized by different factors to introduce varying magnitude of DE (Details in Supplementary Materials). In these scenarios, both scKWARN and SCnorm consistently outperformed other methods, attaining higher F1 scores and lower RMSE values (Supplementary Fig. S5).

**Figure 5.**
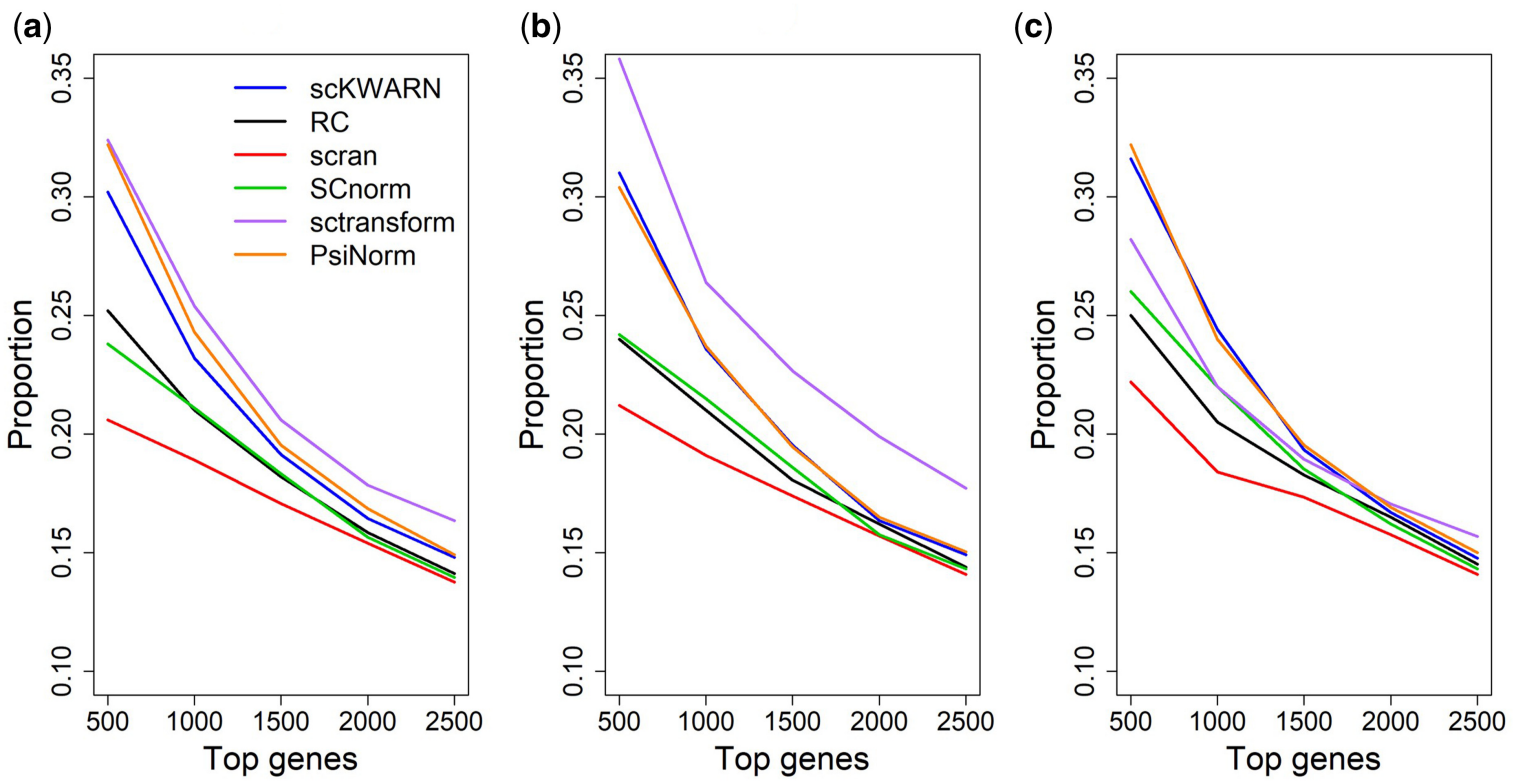
Performance comparison on the GSE29087 dataset. Y axis denotes the proportion of 718 gold standard genes in the top N genes (x axis). The performance was compared in three settings, where none of cells (a), half of the cells (b), or all cells (c) were downsized.

The second dataset was downloaded from the Gene Expression Omnibus (GEO) database with Accession No. GSE29087, comprising 92 cells (48 mouse embryonic cells and 44 mouse embryonic fibroblasts) and 22 928 genes. After filtering out genes expressed in fewer than 3 cells in either of the two groups, 7896 genes were retained for the differential analysis. Among these 7896 genes, 718, which had been validated through qRT-PCR experiments, were considered as the gold standard DE genes (Moliner *et al.* 2008). Since the gold standard genes represent only a subset of true DE genes, as mentioned by (Wang *et al.* 2019), we estimated the proportion of the gold standard genes among the top N genes ranked by each method. The performance was compared in three settings: none of the cells downsized, half of the cells downsized, or all cells downsized by random variables from Uniform(0.2, 1). In the original dataset (no downsizing of cells) or when half of the cells were randomly chosen for downsizing, sctransform achieved the highest accuracy in the detection of DE genes, followed by PsiNorm and scKWARN (Fig. 5a and b). Surprisingly, scran exhibited the lowest proportion, likely attributed to the limited number of cells, which may not be adequate for pooling together to achieve an accurate normalization factor estimation. When all cells were downsized, scKWARN and PsiNorm maintained their performance, while the proportion estimated by sctransform significantly dropped, resulting in the superior performance of scKWARN and PsiNorm over sctransform (Fig. 5c). This result suggests that scKWARN and PsiNorm demonstrate robustness even in scenarios with a high dropout rate and a small number of cells.

**Figure 6.**
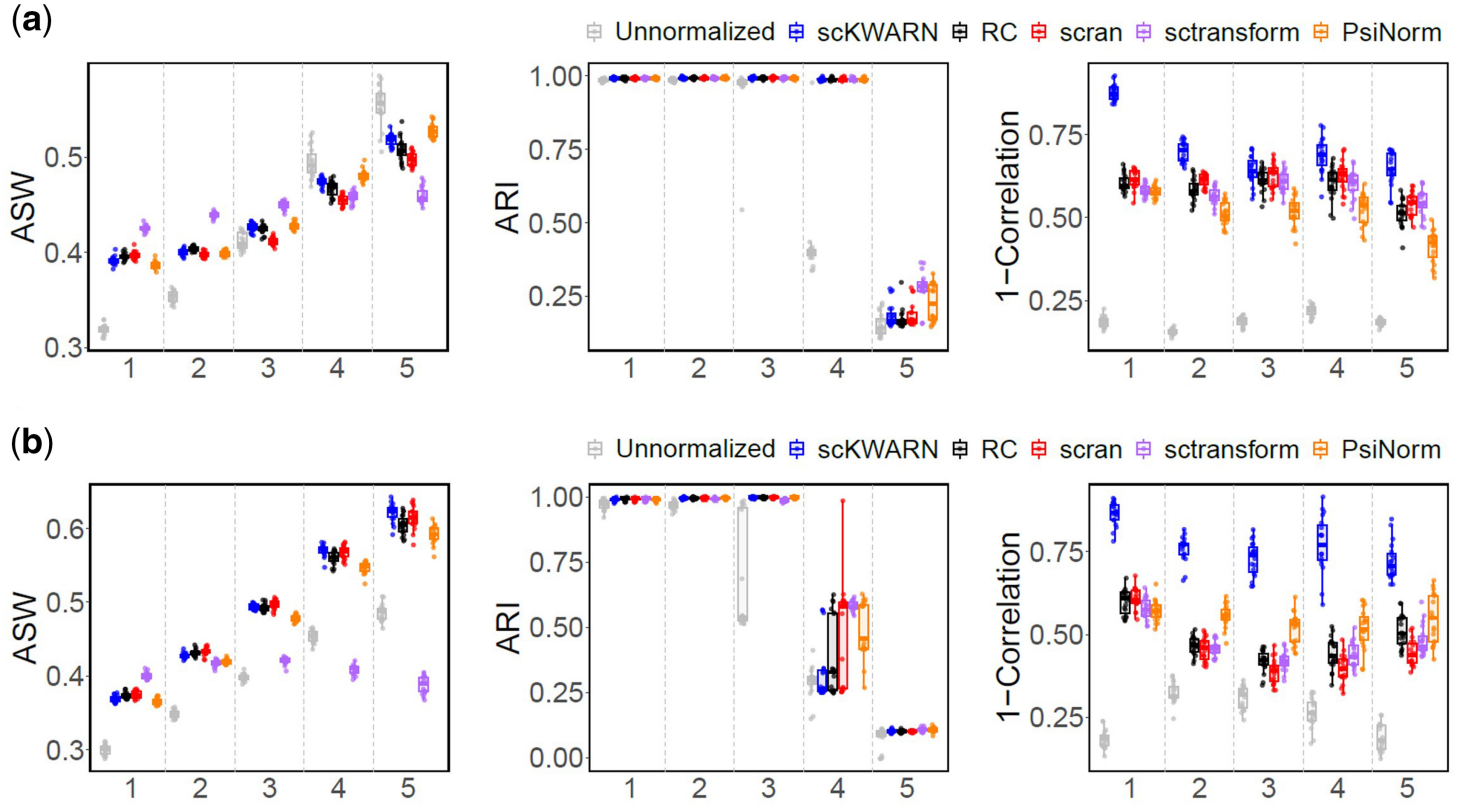
Performance comparison of unnormalized and five normalization methods on the GSE118767 datasets with five scenarios (1–5) where the cell composition changes from balanced (scenario 1) to extremely imbalanced (scenario 5). (a) The imbalanced cell composition was driven by the increasing number of A549 but decreasing of other cell lines. (b) The imbalanced cell composition was driven by the increasing number of H838 but decreasing of other cell lines.

Furthermore, we assessed the clustering performance of scKWARN using the third dataset, GSE118767, which includes ground truth cell type labels. This dataset, generated by various sequencing platforms, comprises seven datasets from five lung adenocarcinoma cell lines: H1975, H2228, HCC827, H838, and A549. Clustering quality was evaluated using Average Silhouette Width (ASW) and Adjusted Rand Index (ARI) (Details in Supplementary Materials). A higher score in these metrics suggests better clustering performance. Additionally, we employed the 1-correlation, where the correlation represents the maximum correlation between PC1 and PC2 of gene expressions and cell sequencing depths (Borella *et al.* 2021). A higher score in this context indicates lower correlation and better performance, signifying that normalization successfully eliminates biases introduced by sequencing depths. The average values of ASW, ARI and 1-correlation across the seven datasets were listed in Supplementary Table S1. Overall, all normalization methods enhanced clustering performance, yielding higher ASW and ARI values compared to unnormalized data. Among normalization methods, SCnorm exhibited the lowest ASW and ARI values and the second lowest 1-correlation score. This result suggests that normalization methods generally perform well in high-quality data. Subsequently, we simulated five scenarios ranging from well-balanced (1:1:1:1:1) to extremely imbalanced cell compositions (36:1:1:1:1) (Supplementary Fig. S6). In balanced settings, sctransform achieved the highest ASW values, while scKWARN and PsiNorm demonstrated higher ASW values as the cell composition became more imbalanced (Fig. 6a and b, UMAP and PCA plots in Supplementary Figs S7 and S8). This trend became more pronounced when the imbalanced cell composition was driven by an increasing number of H838 cells and a decrease in other cell lines (Fig. 6b and Supplementary Fig. S8). All methods yielded higher ARI values than unnormalized data, indicating improved clustering performance (Fig. 6a and b). scKWARN consistently achieved the highest 1-correlation scores in all scenarios (Fig. 6a and b). SCnorm was not included in these scenarios due to lengthy computational time.

### 3.3 Computational performance

We compared the computational performance of the six normalization methods using both real and simulated datasets with varying number of genes and cells (Supplementary Fig. S9). As expected, RC and PsiNorm were the fastest due to simple algorithms. scran and scKWARN required more time than RC and PsiNorm. Sctransform and SCnorm, which depend on model fitting, had the longest computational time. SCnorm even failed on some large datasets.

## 4 Discussion

Accurate normalization is essential for the success of scRNAseq data analysis. An effective normalization method should preserve intrinsic biological features while correcting unwanted biases and be robust against known or hidden technical noises. Here, we introduce scKWARN, a method that does not assume specific data distributions or count-depth relationships. scKWARN has demonstrated improved performance over existing methods in both simulation and real studies. The absence of assumptions about count-depth relationships or read count distributions allows scKWARN to work effectively across diverse settings.

The key strength of scKWARN lies in its way to construct pseudo-profiles for each cell by leveraging information from its fuzzy technical neighbors. These technical neighbors are approximately defined based on the quantile distribution of non-zero profiles. This approach empowers scKWARN to effectively eliminate noise introduced by hidden or unknown factors that impact non-zero counts distributions. Furthermore, each cell has its own reference profile for comparison, ensuring the robustness of the non-DE assumption even in scenarios with unbalanced cell population sizes, a situation frequently overlooked by existing methods.

scKWARN utilizes the 25th, 50th, and 75th percentiles of non-zero profiles to define technical neighbors. These three percentiles are commonly employed to represent the location and scale of data, and they are robust against extreme values. This choice allows scKWARN to capture the main features of the data and counteract the effects of differentially expressed genes and highly expressed genes. While other percentiles between the 25th and 75th are feasible and yield similar results in downstream analysis (not shown), lower or higher percentiles, such as the 10th or 90th percentiles, are not recommended due to large variability in the tails of the expression profile. The use of lower/higher percentiles can result in high RMSE values and sensitive outcomes in differential expression analysis.

scKWARN only employs the first principal component (PC) of the three-dimensional percentile data to identify technical neighbors. This decision was made due to the observation that incorporating additional PCs significantly escalated computational demands without corresponding performance improvements. For instance, employing two PCs (PC1+PC2) led to a 38% increase in computational time compared to using PC1 alone. Nevertheless, scKWARN utilizing PC1+PC2 yielded nearly identical RMSE and F1 scores as the one employing the PC1 alone across the three simulation settings with five different scenarios (Supplementary Tables S2 and S3). The lack of improvement in performance may be attributed to the fact that PC1 already encompasses a substantial amount of variance in the three-dimensional percentile data. For example, PC1 included at least 70% of the variance in the percentile data in those simulations. Moreover, employing additional PCs might even compromise the performance. If more PCs were utilized, there is a greater likelihood of encompassing biological rather than technical variances, introducing potential biases in the determination of technical neighbors.

scKWARN applies a both cell- and gene-specific weighting approach, which can be further improved for better computational performance by incorporating a cutoff option. This option sets low weights to zero when cell distances exceed the bandwidth by a specified cutoff factor (default cutoff = 2). The inclusion of the cutoff option has resulted in approximately 20% savings in computational time while maintaining consistent performance (Supplementary Tables S4 and S5).

## Supplementary Material

btae008_Supplementary_Data

## Data Availability

The data underlying this article are available at https://github.com/cyhsuTN/scKWARN.
