# Supplementary material for: scKWARN: Kernel-weighted-average robust normalization for single-cell RNA-seq data
Source: Bioinformatics. 2024 Jan 17;40(2):btae008. doi: 10.1093/bioinformatics/btae008 (PMC10868328; doi:10.1093/bioinformatics/btae008)
Supplement: btae008_Supplementary_Data [file btae008_supplementary_data.docx]

**Supplementary Materials**

**scKWARN: Kernel-weighted-average robust normalization for single-cell RNA-seq data**

Chih-Yuan Hsu^1,2^, Chia-Jung Chang^1,2,3^, Qi Liu^1,2*^, Yu Shyr^1,2*^

^1^ Department of Biostatistics, Vanderbilt University Medical Center, Nashville, TN 37203

^2^ Center for Quantitative Sciences, Vanderbilt University Medical Center, Nashville, TN 37203

^3^ Department of Biomedical Engineering, National Cheng Kung University, Tainan 701, Taiwan

*Correspondence: [qi.liu@vumc.org](mailto:qi.liu@vumc.org); [yu.shyr@vumc.org](mailto:yu.shyr@vumc.org)

**1. Pseudocode of scKWARN**

**2. Evaluation of Methods**

**3. Simulation Settings**

**4. Real Datasets**

**5. Supplementary Tables and Figures:**

1. Table S1
2. Table S2
3. Table S3
4. Table S4
5. Table S5
6. Figure S1
7. Figure S2
8. Figure S3
9. Figure S4
10. Figure S5
11. Figure S6
12. Figure S7
13. Figure S8
14. Figure S9

**Pseudocode of scKWARN**

**Input:** count data*,* $y_{gj}$*,* $g=1,\ldots,G$ (genes) and $j=1,\ldots, n$ (cells).
**Output:** scale factor $\theta_{j}$

**for**  $j=1,\ldots, n$ **do**

$R_{j}=\{g: y_{gj} > 0, g= 1,..., G\}$*,* the non-zero gene set of cell $j$

$Q_{j}=[q_{0.25,j},q_{0.50,j},q_{0.75,j}]$ of $\{\log(y_{gj}), g\in R_{j}\}$

**end**

$r=$ PC1($Q=[Q_{1}, Q_{2},...Q_{n}]$)*,* the first principal component of $Q$

**for** $g=1,\ldots,G$ **do**

$C_{g}=\left\{ j: y_{gj} > 0, j= 1,..., n \right\}$*,* the cell set expressing gene $g$

$h_{g}=$ SJ bandwidth of $\left\{ r_{j}, j\in C_{g} \right\}$*,* the bandwidth of gene $g$

**end**

**for** $j=1,\ldots, n$ **do**

# the pseudo profile of cell $j$

$A_{j}=\left( a_{gj}, g\in R_{j} \right)$*,* where $a_{gj}=\exp\left\{ \sum_{j^{'}\in C_{g}} w_{g,\left( j,j^{'} \right)}\log\left( y_{gj^{'}} \right) \right\}$

$w_{g,(j,j^{'})}= D_{g,(j,j^{'})}/\sum_{j^{'}\in C_{g}} D_{g,(j,j^{'})}$*,* and $D_{g,(j,j^{'})}= K\{(r_{j}-r_{j^{'}})/h_{g}\}$

# the reference profile of cell $j$

$M_{j}=(m_{g}, g\in R_{j})$*,* where $m_{g}=\exp\left\{ \sum_{j\in C_{g}} \log\left( y_{gj} \right)/|C_{g}| \right\}$

# the scale factor of cell $j$

$\theta_{j}=\mathrm{median}\{\frac{A_{j}}{M_{j}}\}$

**end**

**Evaluation of Methods**

We evaluated six normalization methods, scKWARN, relative counts (RC), scran, SCnorm, sctransform, and PsiNorm in terms of preserving biological variability and improving clustering performance while removing technical biases.

Five metrics, including Bias, RMSE, Sensitivity, Specificity, and F1 score were used to assess the performance of preserving biological variability. Bias quantifies the differences between the true and the estimated log2-fold-changes, while RMSE calculates the average squared difference between the estimated and the actual log2 fold changes. MAST was used to identify DE genes (Finak et al., 2015). The raw p-values from MAST were adjusted by Benjamini-Hochberg procedure and genes with the adjusted p-values < 0.05 were considered to be differentially expressed. Sensitivity is defined as the proportion of the true DE genes which are correctly identified as DE genes, while specificity refers to the proportion of the true non-DE genes which are correctly identified as non-DE genes. F1 score is the harmonic mean of precision and sensitivity.

Two metrics, ASW (the average silhouette width) and ARI (Adjusted Rand Index), were used to estimate the clustering quality. ASW quantifies the expression coherence within the cell type and the separation between different cell types. ARI estimates the agreement between the clusters defined by normalization methods and the ground truth labels. The higher ASW and ARI values suggest the better performance. Cell clustering was carried out using the standard workflow of Seurat, which includes RunPCA for principal component analysis, FindNeighbors for identifying cell neighbors, and FindClusters for grouping cells into clusters.

The correlation index, defined by the maximum correlation between PC1 and PC2 and cell sequencing depths, was originally used by PsiNorm (Borella et al., 2021). The lower correlation suggests better performance, that is, the normalization removes the biases introduced by sequencing depths successfully. Here we used 1-correlation, where the higher score indicates the better performance.

**Simulation settings**

100 simulated datasets were generated for each scenario in three settings. Each simulated data consisted of 3,000 genes and three cell groups. In the three scenarios of simulating the effect of technical noise from library size, RNA composition and dropout rate, the three cell groups were balanced with 200 cells in each population. In the last two scenarios of altering cell population compositions and DE level, the three cell groups were unbalanced, which have 50, 200, and 50 cells.

**Simulation SIM I**

Data generated from linear regression model with normal distribution (Bacher et al., 2017): $\log\left( y_{gj}+1 \right)=\left( 1-D_{gj} \right)\times\left( \log\left( a_{g} \right)+\beta_{0g}+\beta_{1g}\log\left( U_{j} \right)+\varepsilon_{gj} \right)$, where the dropout rate $D_{gj}$ ~ Bernoulli(0.4), $\beta_{0g}$ ~ –7$\times$Gamma(shape = 6, rate = 6) – 8, $\beta_{1g}$ ~ Uniform(0.9, 1.1), $\varepsilon_{gj}$ ~ N(0,$\sigma_{g}^{2}$) with $\sigma_{g}^{2}$ ~ Gamma(shape = 2, rate = 4), and $U_{j}={10}^{6}$. We set $a_{g}=1$, i.e., $\log\left( a_{g} \right)=0$, for all the non-DE genes in each cell group, and $a_{g}$ ~ {2, 3} with P($a_{g}=2$) = 0.8 and P($a_{g}=3$) = 0.2 for the DE genes. In the scenario of altering library sizes of cells, the library size of each cell was upsized or downsized by multiplying a random variable $c_{j}$ from a Uniform(0.5, 5). That was almost equivalent to setting $\beta_{0g}+\log\left( c_{j} \right)$. In the scenario of RNA compositions, 5% genes which were chosen arbitrarily and exclusive of the DE genes were considered as highly expressed genes by setting $\beta_{0g}$ + 3 for all cells. The 5% highly expressed genes dominated library sizes with 20% ~ 60%. In the scenario of dropout rates, we increased the dropout rate with an extra probability 10% ~ 30% by setting $D_{gj}$ ~ Bernoulli(0.4 + $\pi_{gj}$), where $\pi_{gj} \sim$Uniform(0.1, 0.3). In the scenario of cell population compositions, we considered the scenario of altering library sizes in unbalanced populations. In the scenario of the DE level, we considered the scenario of altering library sizes in unbalanced populations with higher DE level from 5% to 15%.

**Simulation SIM II**

Data generated from NB distributions with a linear relationship between mean and count-depth (Hafemeister and Satija, 2019)*:* $y_{gj}$ was generated from a negative binomial distribution with mean $\mu_{gj}=\lambda_{g}\phi_{g}$ and the dispersion parameter value of 0.1, where $\log\left( \lambda_{g} \right)=\beta_{0g}+\beta_{1g}\log_{10}\left( U_{j} \right)$ with $\beta_{0g}$ ~ Uniform(-7, -5), $\beta_{1g}$ ~ Uniform(0.9, 1.1) and $U_{j}={10}^{6}$ for all $j$. Also, $\phi_{g}=1$ for all the genes except the DE genes in each of the three cell groups with $\phi_{g}= 8p$, where $p$ ~ Bernoulli(0.7). In the scenario of altering library sizes of cells, we assumed $\log_{10}\left( U_{j} \right)$ ~ Normal(6, 0.04), and thus $\lambda_{g}(=\lambda_{gj})$ depends on $j$. In the scenario of RNA compositions, 5% genes which are chosen arbitrarily and exclusive of the DE genes were considered as highly expressed genes by multiplying a positive variable from {12, 13, …, 20}. The 5% highly expressed genes dominated 20% ~ 60% of library sizes. In the scenario of dropout rates, we increased the dropout rate with an extra probability 10% ~ 30% by letting $(1-D_{gj})y_{gj}$, where $D_{gj} \sim\mathrm{Bernoulli}(\pi_{gj})$ and $\pi_{gj} \sim$Uniform(0.1, 0.3). In the scenarios of cell population compositions and the DE level, the settings were the same as described in Simulation SIM I.

**Simulation SIM III**

Data generated from NB distributions (Lun et al., 2016): $y_{gj}$ was generated from a negative binomial distribution with mean $\mu_{gj}=\lambda_{g}\phi_{g}$ and the dispersion parameter value of 0.1, where $\lambda_{g}$ ~ Gamma(shape = 2, rate = 2) and $\phi_{g}=1$ for all the genes except the DE genes in each of the three cell groups with $\phi_{g}= 8p$, where $p$ ~ Bernoulli(0.7). In the scenario of altering library sizes of cells, the mean parameters of NB distributions were set to $\mu_{gj}=\lambda_{g}\phi_{g}c_{j}$, where $c_{j}$ ~ Uniform(0.5, 2). In the scenario of RNA compositions, 5% genes which were chosen arbitrarily and exclusive of the DE genes were considered as highly expressed genes by multiplying a positive variable from {12, 13, …, 20} with the equal probability of 1/9. The 5% highly expressed genes dominated 20% ~ 60% of library sizes. In the scenario of dropout rates, we increased the dropout rate with an extra probability 10% ~ 30% by letting $(1-D_{gj})y_{gj}$, where $D_{gj} \sim\mathrm{Bernoulli}(\pi_{gj})$ and $\pi_{gj} \sim$Uniform(0.1, 0.3). In the scenarios of cell population compositions and the DE level, the settings were the same as described in Simulation SIM I.

**Real Datasets**

**PBMC 33K case study**

The first real dataset, PBMC33K, from a Healthy Donor, v1 Chemistry 10x Genomics consists of 33,148 human peripheral blood mononuclear cells and 32,738 genes. The dataset is available at <https://support.10xgenomics.com/single-cell-gene-expression/datasets/1.1.0/pbmc33k>. Two cell groups were generated from the duplicated 1,000 cells randomly sampled from the B cells (with highly expressed CD79A gene). Then, 10% of genes in one cell group were randomly selected and were upsized by a variable sampled from {1.25, 1.5, …, 29.75, 30} with equal probability, while 10% of genes in the other cell group randomly selected and exclusive of the genes selected in the first group and were upsized by a variable sampled from {1.25, 1.5, …, 4.75, 5}. In the scenario of strong DE, 25% of genes were upsized for each group. In the unbalanced setting, one cell group had 1,000 cells, while the other only included 100 cells. Additionally, we introduced four scenarios involving downsizing of genes. In these scenarios, the expressions of selected genes were downsized by a variable sampled from {0.1, 0.2, …, 0.8, 0.9} with equal probability.

**GSE29087 case study**

The second real dataset from the Gene Expression Omnibus (GEO) database with Accession No. GSE29087 consists of 92 cells (48 mouse embryonic cells and 44 mouse embryonic fibroblasts) and 22,928 genes, available at [https://www.ncbi.nlm.nih.gov/geo/query/acc.cgi?acc=GSE29087](https://nam05.safelinks.protection.outlook.com/?url=https%3A%2F%2Fwww.ncbi.nlm.nih.gov%2Fgeo%2Fquery%2Facc.cgi%3Facc%3DGSE29087&data=02%7C01%7Cchih-yuan.hsu%40vumc.org%7C39cba746963944f98fa308d80be7f8c7%7Cef57503014244ed8b83c12c533d879ab%7C0%7C0%7C637272438218351330&sdata=mGMHNMcV012B82xspE2%2F%2Bpu85VSnwHuapD%2FKyZI2hBQ%3D&reserved=0). We filtered genes which were expressed in less than 3 cells in either of the two groups, where 7,896 genes were kept for the downstream analysis. Of the 7,896 genes, 718 genes were considered as the gold standard DE genes according to the top 1000 DE genes in (Moliner et al., 2008).

**GSE118767 case study**

The third data were downloaded from GEO with Accession No. GSE118767, which consist of seven datasets generated by different platforms, mix.CELSeq51, mix.CELSeq52, mix.CELSeq53, mix.DropSeq, mix.10x, mix.10x5, and mix.CELSeq. They include five lung adenocarcinoma cell lines, H1975, H2228, HCC827, H838, and A549. The data are available at <https://www.ncbi.nlm.nih.gov/geo/query/acc.cgi?acc=GSE118767>.

Among them, the mix.10x5 dataset had the greatest number of cells, which were used to generate five scenarios ranging from well-balanced (scenario 1, 1:1:1:1:1) to extremely imbalanced cell compositions (scenario 5: 36:1:1:1:1). The number of five cell types in each scenario was shown in Supplementary Figure S6. Two settings were simulated. In one setting, the imbalanced cell composition was driven by the increasing number of A549 but decreasing of other cell lines (Supplementary Figure S6a). In the other setting, the imbalanced cell composition was driven by the increasing number of H838 but decreasing of other cell lines (Supplementary Figure S6b).

**Table S1.** The performance comparison on the seven datasets of GSE118767.

|  | **Unnormalized** | **scKWARN** | **RC** | **scran** | **SCnorm** | **sctransform** | **PsiNorm** |
| --- | --- | --- | --- | --- | --- | --- | --- |
| **ASW** | 0.2343 | 0.2643 | 0.2688 | 0.2682 | 0.2568 | 0.3006 | 0.2646 |
| **ARI** | 0.8864 | 0.9780 | 0.9823 | 0.9733 | 0.9434 | 0.9799 | 0.9668 |
| **1-Correlation** | 0.1769 | 0.6436 | 0.7043 | 0.7609 | 0.5651 | 0.7049 | 0.5021 |

Unnormalized: data is not normalized.

**Table S2.** RMSE (standard deviation) obtained by scKWARN using PC1 or PC1+PC2 in the three simulation settings with five different scenarios (100 simulations).

|  | scKWARN | S1 | S2 | S3 | S4 | S5 |
| --- | --- | --- | --- | --- | --- | --- |
| SIM I | PC1 | 0.034 (0.004) | 0.002 (0.001) | 0.003 (0.001) | 0.035 (0.005) | 0.036 (0.006) |
|  | PC1 + PC2 | 0.032 (0.003) | 0.002 (0.001) | 0.003 (0.001) | 0.033 (0.005) | 0.034 (0.005) |
| SIM II | PC1 | 0.443 (0.006) | 0.013 (0.002) | 0.019 (0.003) | 0.728 (0.013) | 0.759 (0.017) |
|  | PC1 + PC2 | 0.443 (0.006) | 0.015 (0.002) | 0.019 (0.003) | 0.727 (0.013) | 0.759 (0.017) |
| SIM III | PC1 | 0.439 (0.006) | 0.015 (0.002) | 0.020 (0.003) | 0.720 (0.016) | 0.754 (0.014) |
|  | PC1 + PC2 | 0.439 (0.006) | 0.015 (0.002) | 0.021 (0.003) | 0.720 (0.016) | 0.754 (0.014) |

S1: Library size, S2: RNA composition, S3: Dropout rate, S4: Cell composition, S5: Cell composition + Strong DE

**Table S3.** F1 scores (standard deviation) obtained by scKWARN using PC1 or PC1+PC2 in the three simulation settings with five different scenarios (100 simulations).

|  | scKWARN | S1 | S2 | S3 | S4 | S5 |
| --- | --- | --- | --- | --- | --- | --- |
| SIM I | PC1 | 0.955 (0.013) | 0.954 (0.013) | 0.935 (0.017) | 0.912 (0.021) | 0.938 (0.010) |
|  | PC1 + PC2 | 0.955 (0.012) | 0.954 (0.013) | 0.935 (0.017) | 0.913 (0.020) | 0.938 (0.010) |
| SIM II | PC1 | 0.855 (0.019) | 0.849 (0.020) | 0.700 (0.035) | 0.209 (0.068) | 0.379 (0.041) |
|  | PC1 + PC2 | 0.854 (0.019) | 0.850 (0.020) | 0.700 (0.036) | 0.206 (0.069) | 0.378 (0.041) |
| SIM III | PC1 | 0.843 (0.017) | 0.846 (0.017) | 0.698 (0.033) | 0.203 (0.063) | 0.345 (0.043) |
|  | PC1 + PC2 | 0.844 (0.017) | 0.846 (0.017) | 0.698 (0.033) | 0.201 (0.064) | 0.347 (0.043) |

S1: Library size, S2: RNA composition, S3: Dropout rate, S4: Cell composition, S5: Cell composition + Strong DE

**Table S4.** RMSE (standard deviation) obtained by scKWARN with and without cutoff in the three simulation settings with five different scenarios (100 simulations).

|  | scKWARN | S1 | S2 | S3 | S4 | S5 |
| --- | --- | --- | --- | --- | --- | --- |
| SIM I | No_cutoff | 0.034 (0.004) | 0.002 (0.001) | 0.003 (0.001) | 0.035 (0.005) | 0.036 (0.006) |
|  | Cutoff = 2 | 0.034 (0.004) | 0.002 (0.001) | 0.003 (0.001) | 0.035 (0.005) | 0.036 (0.006) |
| SIM II | No_cutoff | 0.443 (0.006) | 0.013 (0.002) | 0.019 (0.003) | 0.728 (0.013) | 0.759 (0.017) |
|  | Cutoff = 2 | 0.443 (0.006) | 0.013 (0.002) | 0.019 (0.003) | 0.728 (0.013) | 0.759 (0.017) |
| SIM III | No_cutoff | 0.439 (0.006) | 0.015 (0.002) | 0.020 (0.003) | 0.720 (0.016) | 0.754 (0.014) |
|  | Cutoff = 2 | 0.439 (0.006) | 0.015 (0.002) | 0.020 (0.003) | 0.720 (0.016) | 0.754 (0.014) |

S1: Library size, S2: RNA composition, S3: Dropout rate, S4: Cell composition, S5: Cell composition + Strong DE

**Table S5.** F1 scores (standard deviation) obtained by scKWARN with and without cutoff in the three simulation settings with five different scenarios (100 simulations).

|  | scKWARN | S1 | S2 | S3 | S4 | S5 |
| --- | --- | --- | --- | --- | --- | --- |
| SIM I | No_cutoff | 0.955 (0.013) | 0.954 (0.013) | 0.935 (0.017) | 0.912 (0.021) | 0.938 (0.010) |
|  | Cutoff = 2 | 0.955 (0.013) | 0.954 (0.013) | 0.935 (0.017) | 0.913 (0.021) | 0.938 (0.010) |
| SIM II | No_cutoff | 0.855 (0.019) | 0.849 (0.020) | 0.700 (0.035) | 0.209 (0.068) | 0.379 (0.041) |
|  | Cutoff = 2 | 0.855 (0.019) | 0.850 (0.020) | 0.700 (0.035) | 0.209 (0.069) | 0.379 (0.041) |
| SIM III | No_cutoff | 0.843 (0.017) | 0.846 (0.017) | 0.698 (0.033) | 0.203 (0.063) | 0.345 (0.043) |
|  | Cutoff = 2 | 0.843 (0.017) | 0.846 (0.017) | 0.699 (0.032) | 0.202 (0.064) | 0.345 (0.043) |

S1: Library size, S2: RNA composition, S3: Dropout rate, S4: Cell composition, S5: Cell composition + Strong DE


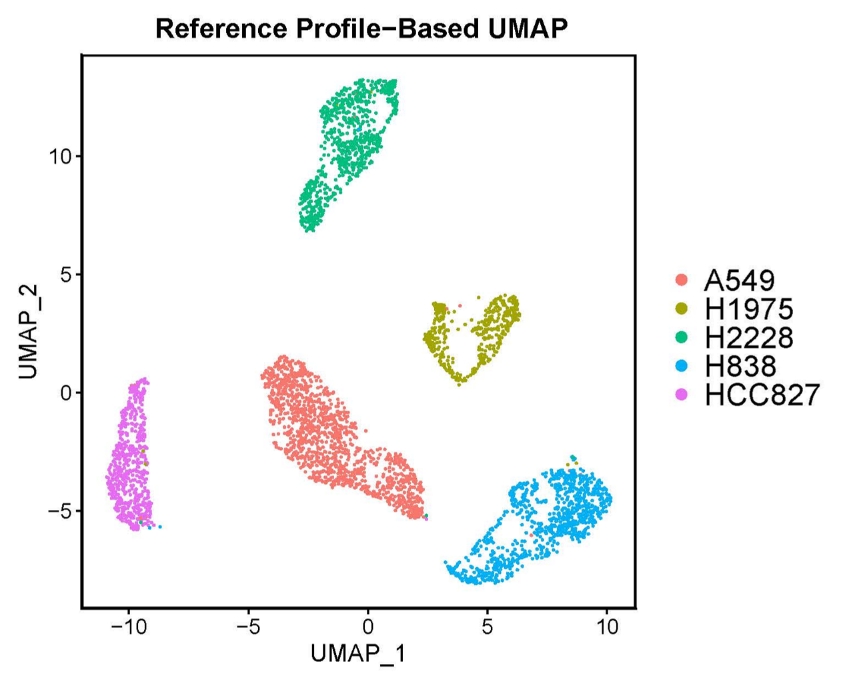


**Figure S1.** UMAP plots of reference profiles of GSE118767 mix10x5, each color representing one cell type.


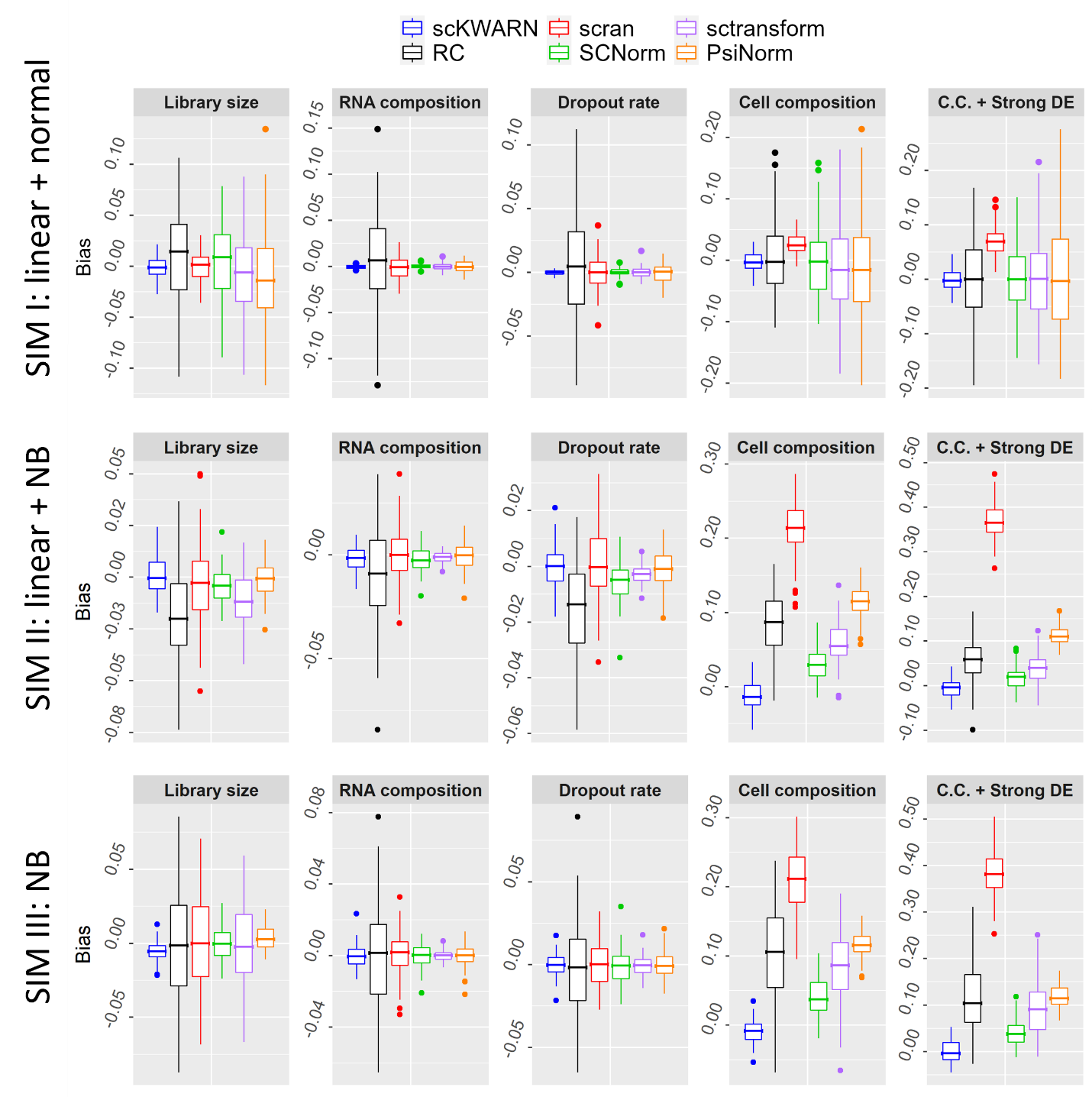


**Figure S2.** Performance comparison in terms of Bias in three simulation settings with five scenarios. Bias quantifies the differences between the true and the estimated log2-fold-changes.


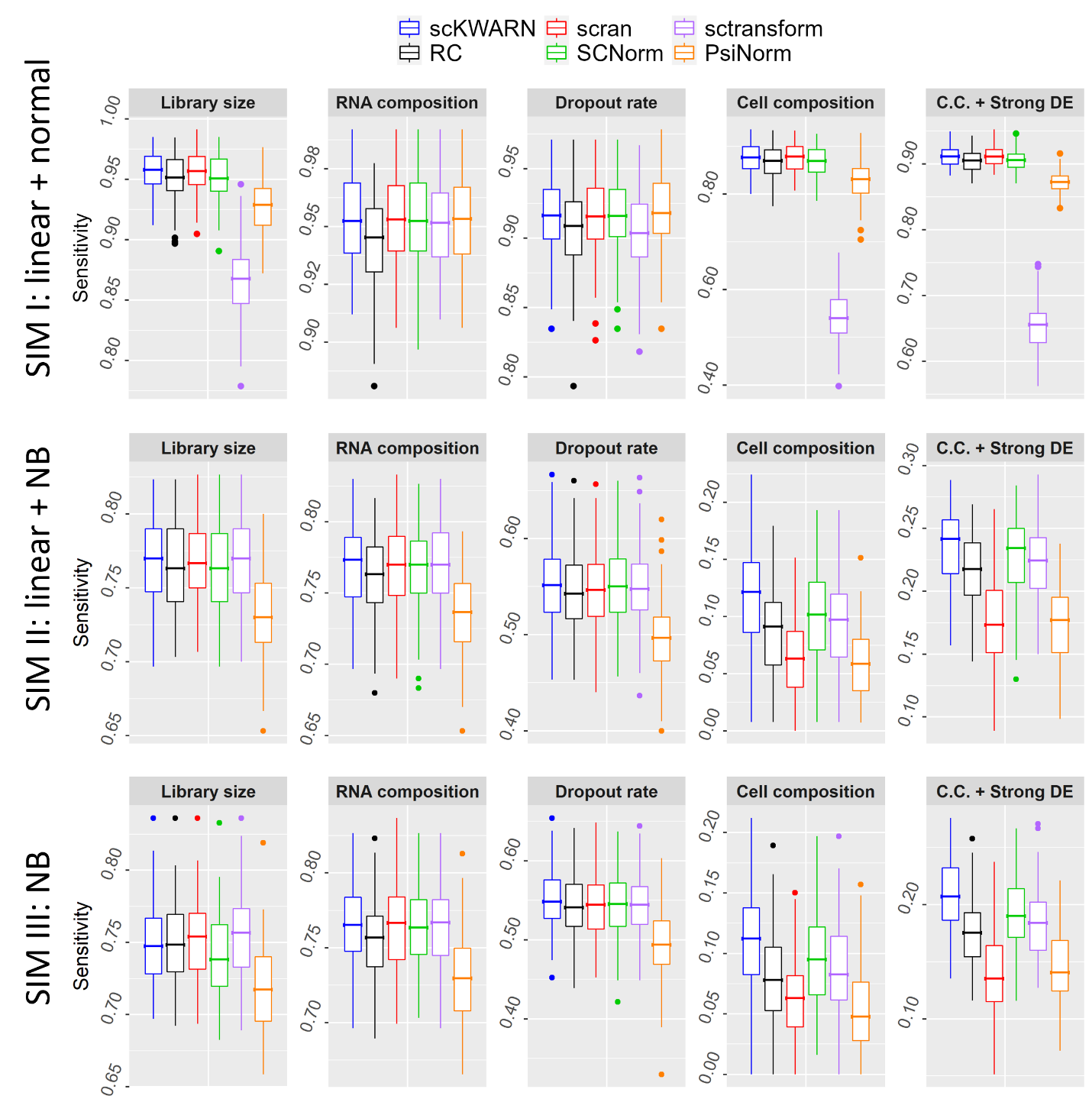


**Figure S3.** Performance comparison in terms of Sensitivity in three simulation settings with five scenarios. Sensitivity is defined as the proportion of the true DE genes which are correctly identified as DE genes.


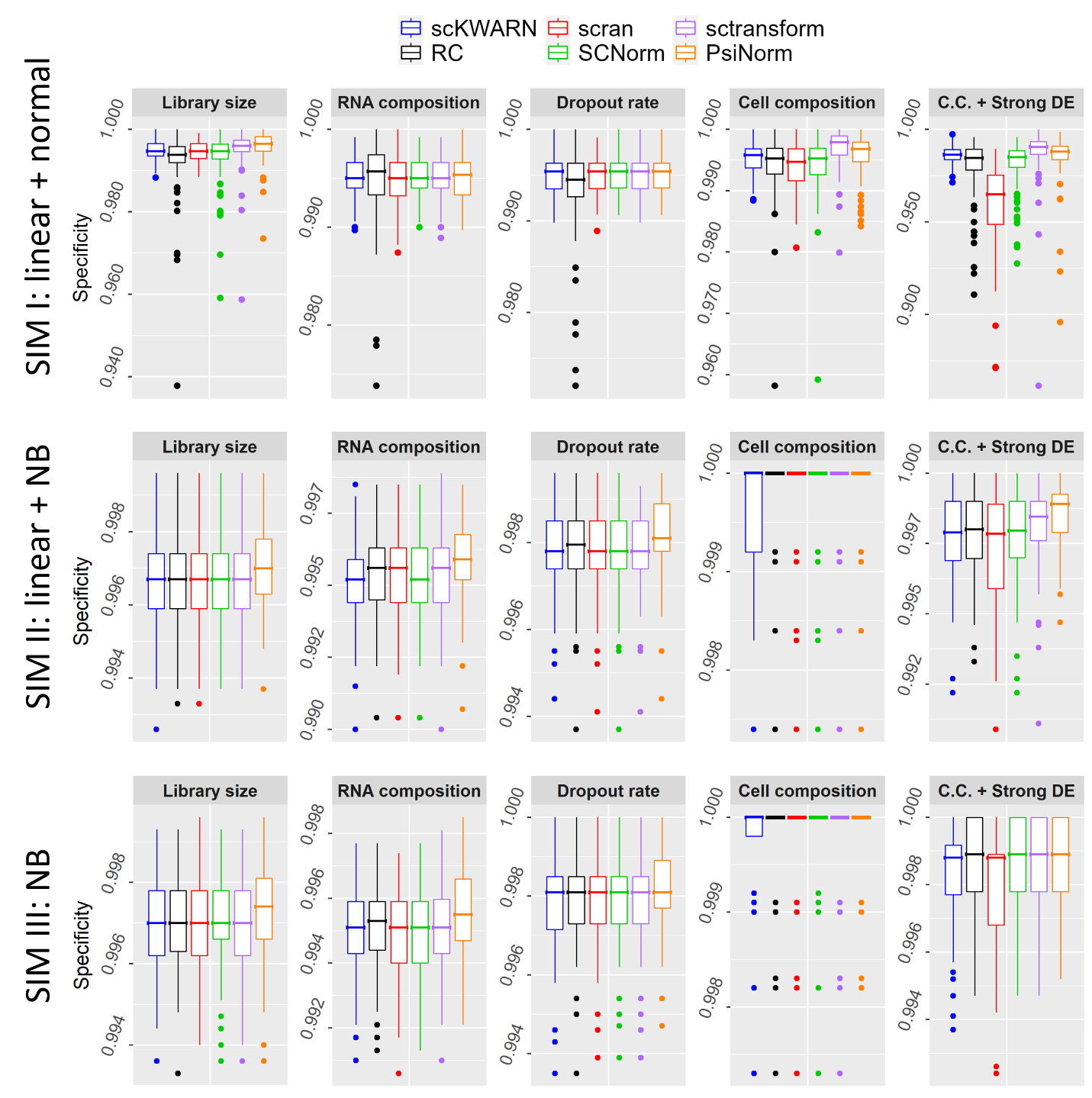


**Figure S4.** Performance comparison in terms of Specificity in three simulation settings with five scenarios. Specificity refers to the proportion of the true non-DE genes which are correctly identified as non-DE genes.


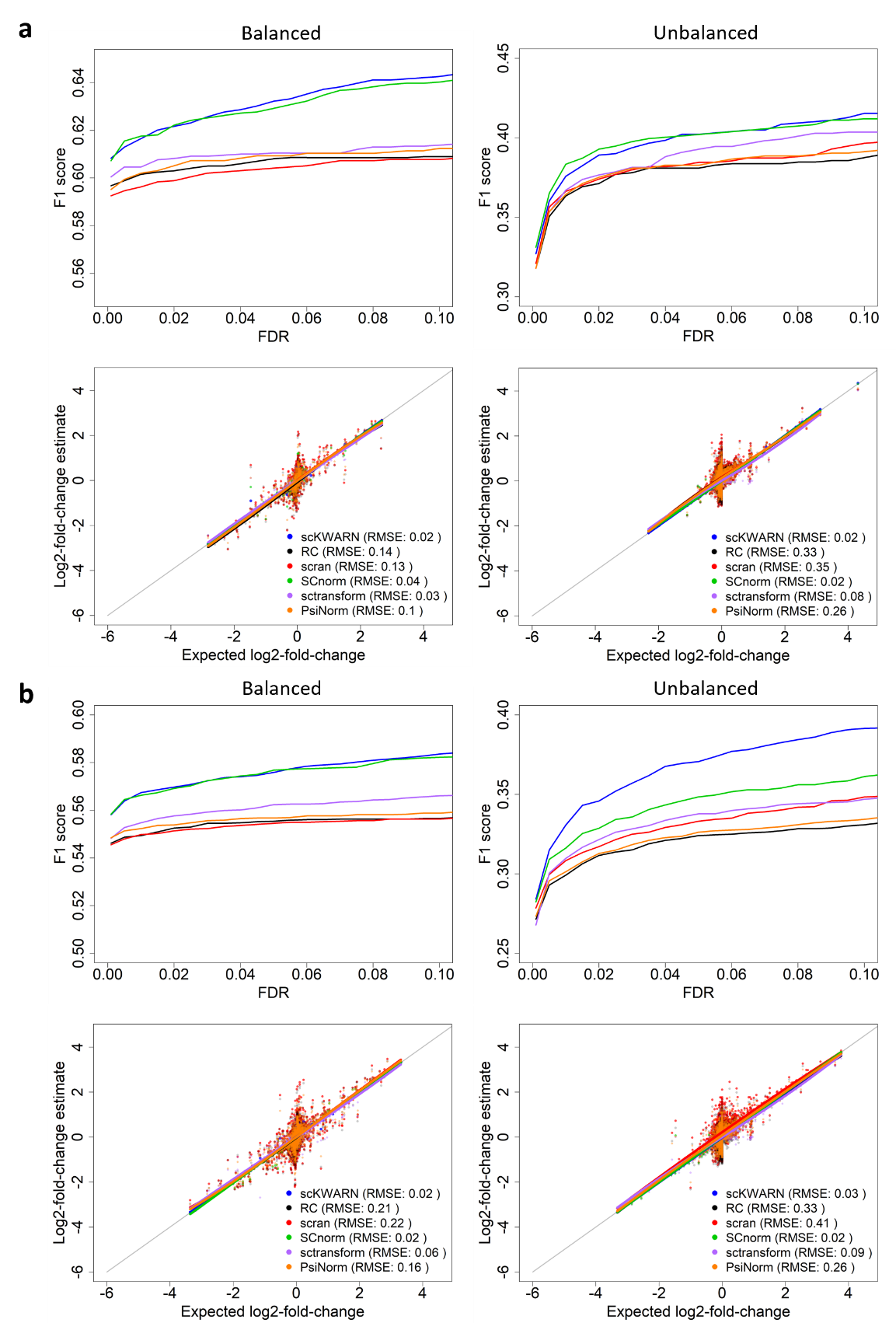


**Figure S5.** Performance comparison on the PBMC33K dataset with Moderate DE (a) and Strong DE (b) settings, where genes were downsized. The balanced setting has each cell population of 1,000 cells (left panel), while the unbalanced includes one of 1,000 and the other of only 100 cells (right panel).


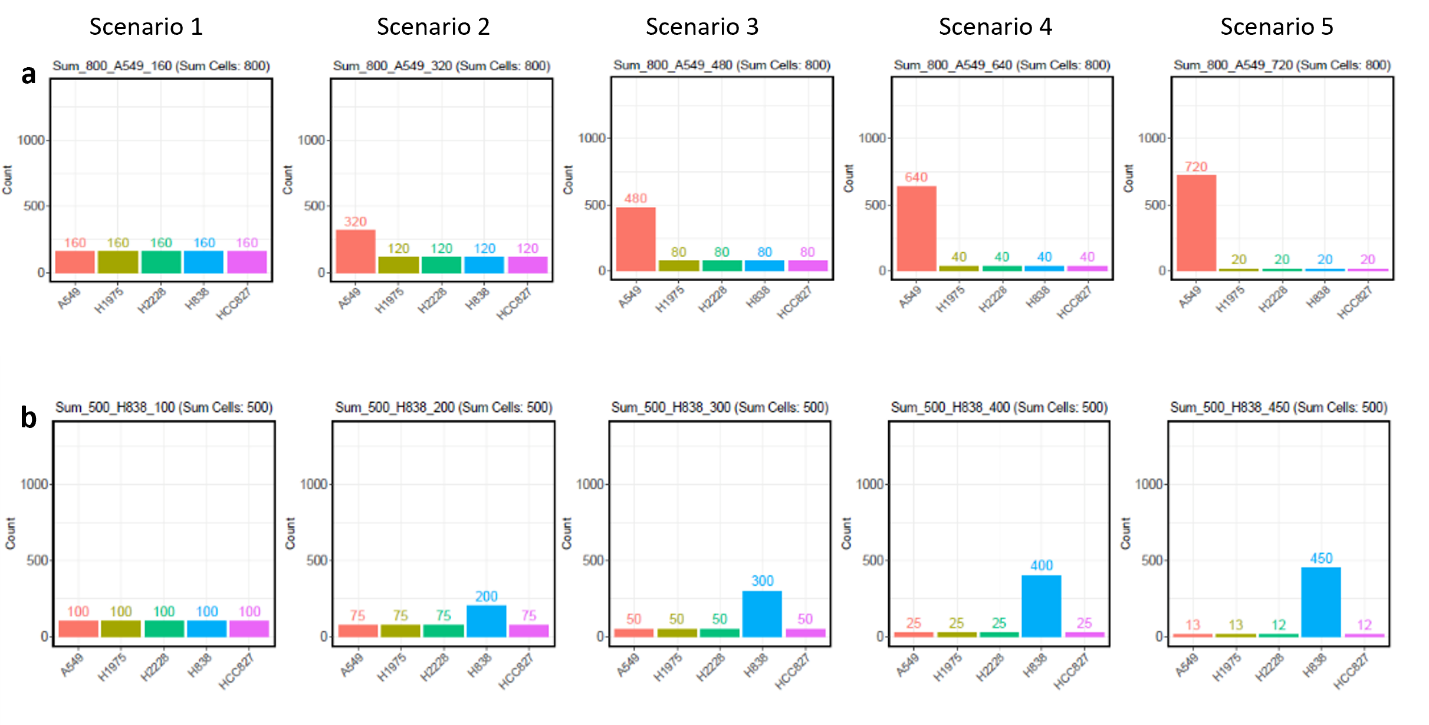


**Figure S6.** The cell composition in the two settings with five scenarios where cell population changes from balanced (scenario 1) to extremely imbalanced (scenario 5). (a) The cell composition change is driven by the increasing number of A549 cell lines but decreasing number of other cell lines. (b) The cell composition change is driven by the increasing number of H838 cell line but decreasing of other cell lines.


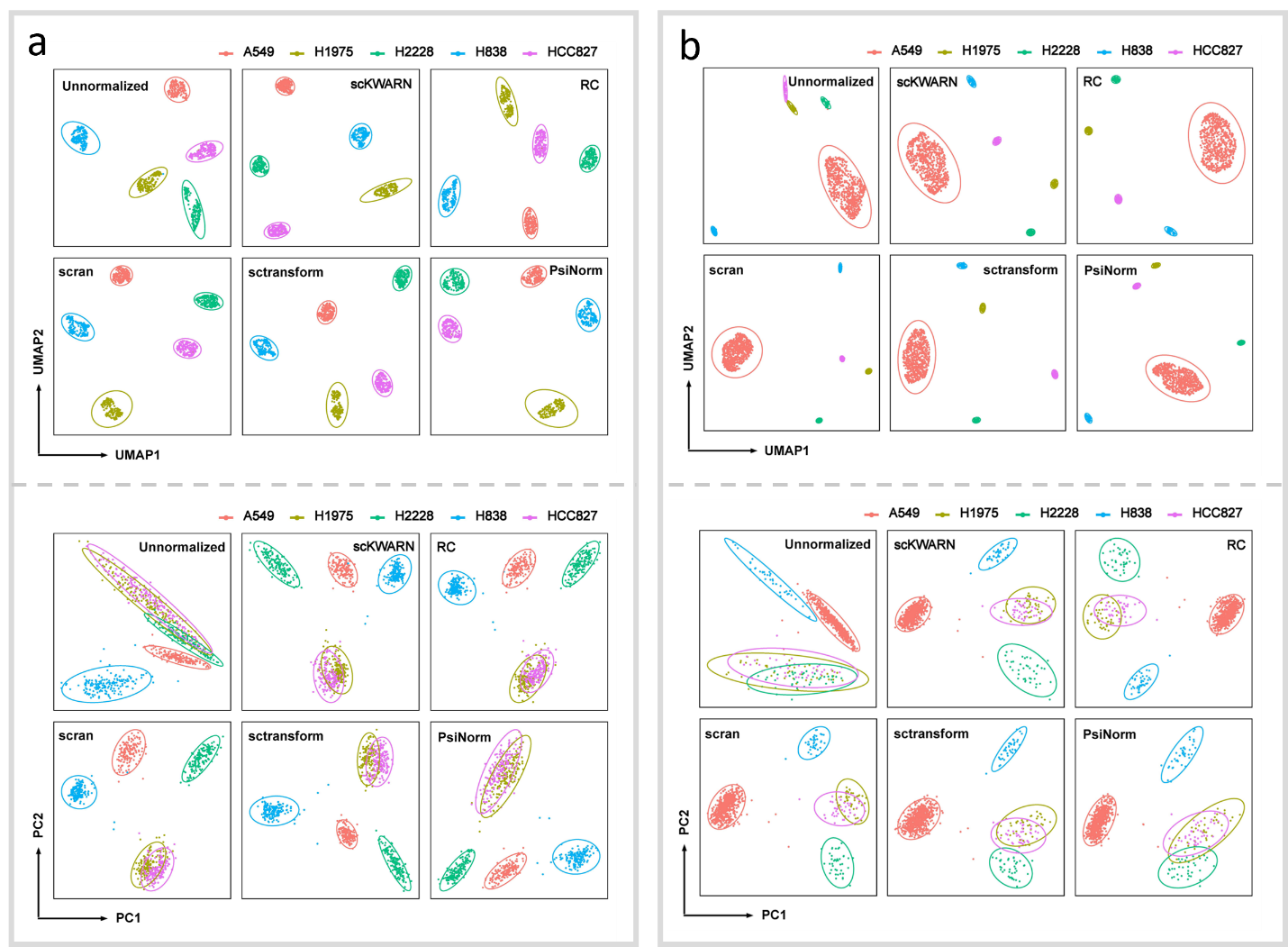


**Figure S7.** UMAP and PCA plots of unnormalized and normalized data using scKWARN, RC, scran, sctransform, and PsiNorm on the GSE118767 datasets in the scenario 1 (a) and 4 (b). In the scenario 1, the cell population is balanced with each of the five cell types having 160 cells (1:1:1:1:1). In the scenario 4, the cell population is very imbalanced with A549 having 640 cells and each of the other four cell types having 40 cells (16:1:1:1:1).


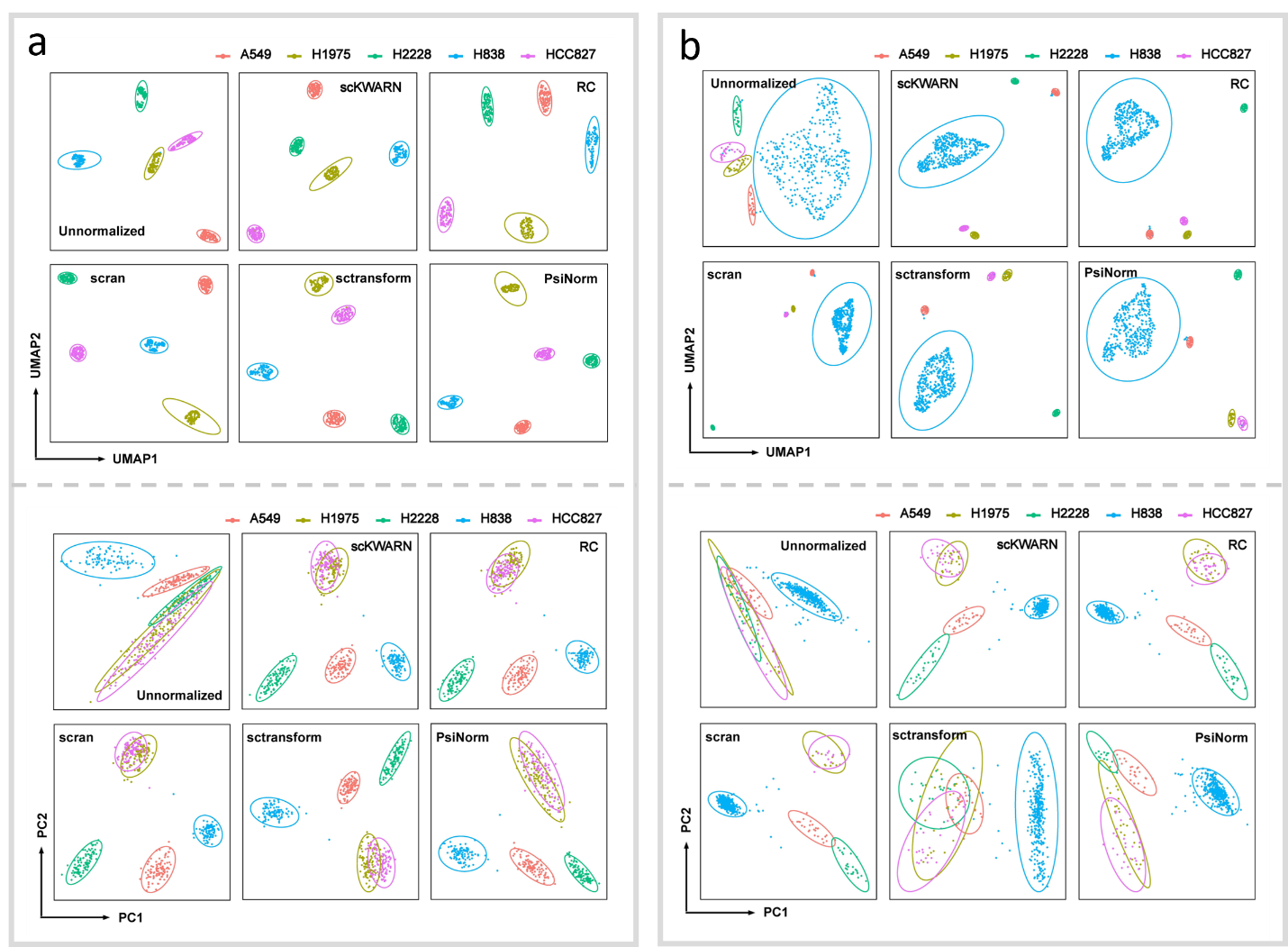


**Figure S8.** UMAP and PCA plots of unnormalized and normalized data using scKWARN, RC, scran, sctransform, and PsiNorm on the GSE118767 datasets in the scenario 1 (a) and 4 (b). In the scenario 1, the cell population is balanced with each of the five cell types having 100 cells (1:1:1:1:1). In the scenario 4, the cell population is very imbalanced with H838 having 450 cells and each of the other four cell types having 25 cells (16:1:1:1:1).


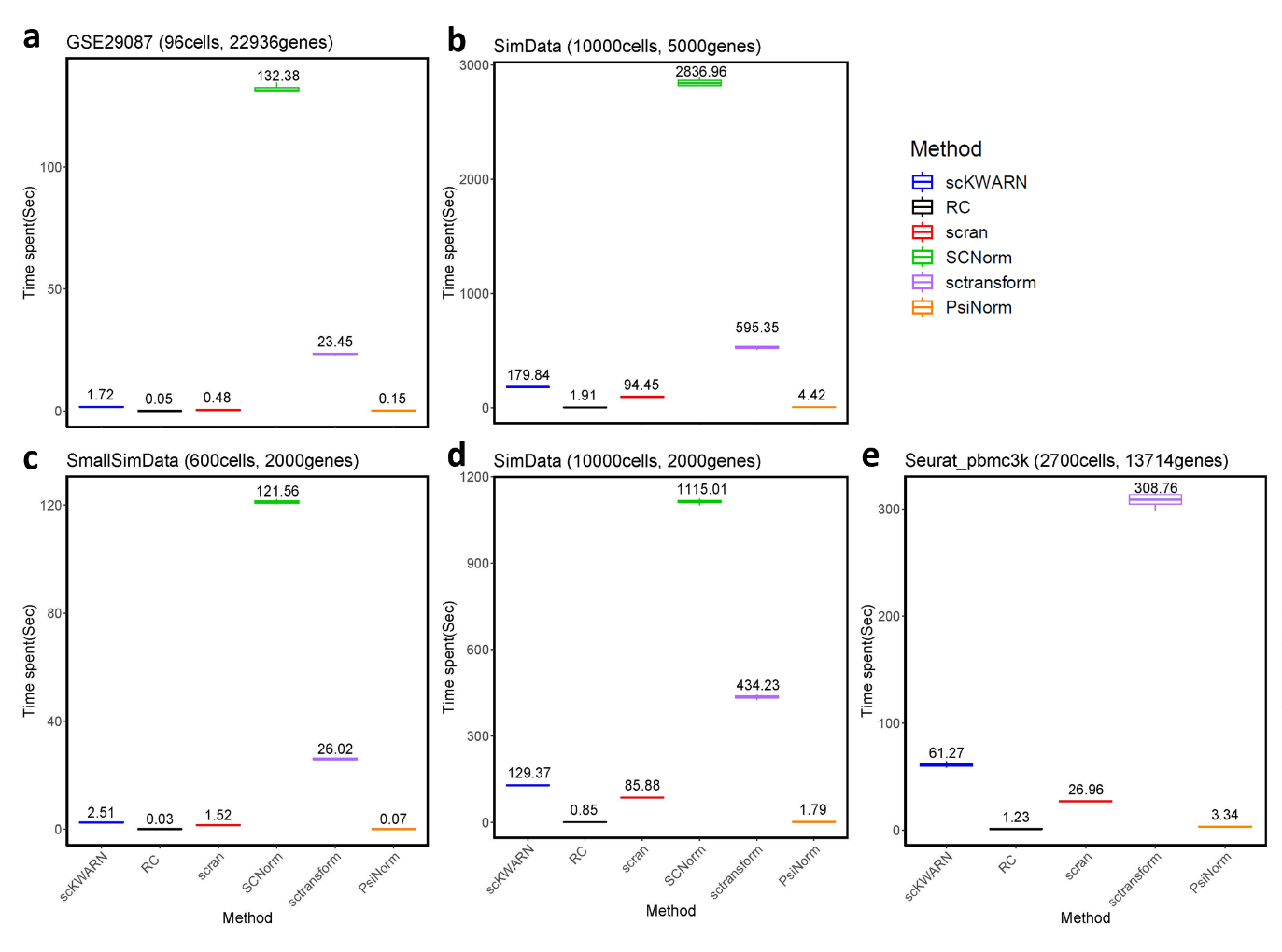


**Figure S9.** Comparison on the Computational performance. Box plot with x-axis representing various normalization methods and y-axis shows time in seconds. (a) Real data from GSE29087. (b)-(d) Simulated data with varying numbers of cells and genes (data were generated from negative binomial distributions). (e) Real data from Seurat pbmc3k.
